# System Pharmacological Approach to Investigate and Validate Multitargeted and Therapeutic Effect of Furocoumarins of *Apium graveolens* L. for Treatment of Kidney Disease

**DOI:** 10.1155/2024/5543561

**Published:** 2024-04-17

**Authors:** Yasheshwar, Gaurav, Rustam Ekkbal, Prem Gupta, Rajendra Gyawali

**Affiliations:** ^1^Department of Botany, Acharya Narendra Dev College (University of Delhi), Govindpuri, Kalkaji, New Delhi 110019, India; ^2^Department of Research and Development, Hamdard Laboratories (Medicine), D-38, Industrial Area, Meerut Road, Ghaziabad 201002, Uttar Pradesh, India; ^3^Modern Diagnostic and Research Centre, 363/4 Jawahar Nagar, Sector-12, Gurugram 122001, Haryana, India; ^4^Department of Pharmacy, Kathmandu University, Dhulikhel, Nepal

## Abstract

**Background:**

System pharmacological approaches play important roles in drug discovery and development and in biomolecular exploration to investigate the multitarget therapeutic effects of phytochemicals for the treatment of acute and chronic ailments.

**Objectives:**

The aim of the study was to apply a system pharmacological approach to investigate the multitarget therapeutic effects of furocoumarins of *Apium graveolens* L. for the treatment of kidney disease.

**Methods:**

Several furocoumarins of *Apium graveolens* were screened from online databases. Network biology and poly-pharmacology analyses were performed to investigate the multitarget therapeutic effect of furocoumarins. The potential metabolites that showed significant interactions with various genes were selected for in silico docking analysis with CASP-3 and SOD proteins. In silico ADME analysis was also performed to investigate the pharmacokinetic behavior of targeted furocoumarins.

**Results:**

Out of thirteen furocoumarins selected for analysis, six showed partial or significant interaction with SOD and CASP-3 proteins. These metabolites may alleviate kidney dysfunction by reducing oxidative and inflammatory stress, regulating apoptosis, slowing down the progression of diabetic nephropathy, and reducing hypertension and glomerular vascular rigidity. In silico docking analysis revealed bergapten as a potential therapeutic agent for kidney disease treatment. In silico docking analysis showed anglicine, imperatorin, and sphondin exhibited strong interaction with CASP-3 and SOD with binding energy −6.5, −7.2, −6.5 and −6.8, −6.2 −5.7 kcal/mol, respectively. These components exhibited greater conventional hydrogen bonding with CASP-3 and SOD than other furocoumarins. Furthermore, in silico ADME analysis of metabolites showed that all furocoumarins have a highly lipophilic nature, good skin permeability, and GI absorption, as well as good blood-brain permeability (BBB).

**Conclusion:**

Furocoumarins reduce kidney dysfunction and associated pathophysiological complications via the reduction of glomerular vascular rigidity, diabetic nephropathy, and oxidative and inflammatory stress. However, further biomolecular and clinical examinations are necessary to validate and enhance the credibility of present findings.

## 1. Introduction

Progressive segmental glomerulitis is one of the characteristics of chronic kidney disease (CKD). The incidence of CKD is rising due to several pathophysiological onsets including oxidative stress, inflammation, diabetes, and hypertension [1]. It has been estimated that the projected global prevalence of kidney disease may be more than the current estimates, and it can become a serious burden in the future for the economy and medical systems of various nations. The age-adjusted incidence rate of ESRD in India has recently been reported to be 229 per million people [2]. The causes of kidney disease and glomerular disorders are diverse and complex. Kidney disease can be caused by a variety of factors, including medication toxicity, ischemia following thoracic operations, and septic infection [3]. Nonsteroidal anti-inflammatory drugs, anticancer medicines, antibiotics, and immunosuppressive drugs are examples of drug toxins that induce kidney disease [4]. Reduced blood and oxygen flow due to systemic hypotension, systemic hypoxia, and altered localized oxygen delivery to the kidney also lead to kidney disease which generally begins with glomerular impairment and later results in kidney damage exterior of the glomerular architecture [5]. Oxidative stress and inflammation induced by the deleterious effect of oxidants cause several pathophysiological changes in the normal function of the kidney [6]. Glomerular mesangial cells, podocytes, and endothelial cells are injured, resulting in glomerulosclerosis and enhanced extracellular matrix deposition, leading to kidney dysfunction [7].

Modern medicine offers several types of drugs which can help in the management of kidney disease [8]. Despite large-scale utilization of these drugs, therapy for kidney disease is far from perfect due to several factors such as the inability to reverse the already damaged kidney function, adverse reactions, cost, and problems of availability and accessibility of drugs.

In recent years, the introduction of multitarget drugs has resulted in innovative treatments that can potentially cure renal ailments. The requirement to optimize medications against many biologic targets while keeping correct pharmacological characteristics is a key problem for creating multitarget pharmaceuticals, also known as various ligand drugs [9]. An additional significant problem in creating multitarget medications is defining the appropriate activity equilibrium and integrating pharmacological characteristics with affinity for biological sites [10]. The use of structural knowledge and structure-based modelling allows for the development of multitarget drugs with specificity for the desired biological targets [11]. In silico approaches contribute significantly to the evaluation of a drug's therapeutic value on the basis of its intensity of ligation with a particular target gene or protein. Furthermore, network pharmacology investigation can throw light on the varied therapeutic properties of a drug molecule and the biological processes affected by it for the treatment of a disease [12].

Network pharmacology investigates how several medicines or phytomolecules interact and influence gene expression [13]. Network-based methodologies are increasingly being employed to investigate the multimechanistic therapeutic activities of phytochemicals [14]. Plants have long played an important role in the traditional medicine systems of developing nations and in local people's cultures and customs. For generations, Indians have recognized medicinal plants as rich sources of therapeutic substances for managing a range of diseases [15]. Several studies carried out in various regions of India reveal that at least 75% of the Indian population use medicinal herbs to meet their healthcare demands [15], due to various reasons, including the rising cost of conventional medicines and lower risk of adverse reactions [16].

*Apium graveolens* L is a medicinal plant that has been used in India since ancient times for its hepatoprotective, immunomodulatory, antitumor, fibrinolytic, hypoglycemic, anti-inflammatory, and nephroprotective properties [17]. The roots and seeds of the plant contain a variety of phytochemicals such as limonene, selinene, coumarin glycosides, flavonoids, and vitamins A and C, which makes it quite useful in traditional medicine [18]. In India, the aqueous extracts of plant roots and seeds have been utilized for medicinal purposes since ancient times [19]. Several investigations on the nephroprotective benefits of *Apium graveolens* L against drug-induced nephrotoxicity have been conducted [20]. However, there is a need to investigate the molecular mechanisms involved in the beneficial effects of this plant on the pathophysiology of renal disease.

## 2. Methodology

### 2.1. Computational Tools Used for the Study

Cytoscape, Metascape, AutoDock, Discovery Studio Visualizer, and SwissADME were used for computational analyses (network biology, network pharmacology, and pharmacokinetic studies).

### 2.2. Selection of Principle Metabolites of *Apium graveolens* L

Literature was screened for studies on the phytochemical components of *Apium graveolens*. Keywords such as phytochemistry of *Apium graveolens*, chemical constituents of *Apium graveolens*, and furocoumarins of *Apium graveolens* were used to explore the constituents. Furocoumarins were finally selected for evaluation of their multimechanistic therapeutic effects in the alleviation of acute and chronic ailments [21]. The furocoumarins phytoconstituents selected for the study are summarized in Table 1.

### 2.3. Network Pharmacology Analysis

Each selected *Apium graveolens* L. metabolite was investigated for probable interactions with various genes implicated in kidney disease. All metabolites and the genes with which they were found to interact were displayed as a network. Edges that were directly associated with the functional metabolites were added to the final evaluation, whereas edges that were not associated with the metabolites were excluded. The potential genes involved in kidney disease were selected from the Gene card (https://www.genecards.org/) and the UniPort database (https://www.uniprot.org/uploadlists/) [22]. Protein-protein interaction (PPI) and compound-protein interaction (CPI) analyses were performed using the STRING platform (https://string-db.org/) and Cytoscape (Version 3.8.2) software. The target PPI was imported to Cytoscape software for integration and further protein-ligand construction analysis. Furthermore, gene ontology analysis was performed to determine the multimechanistic and therapeutic effects of metabolites in the pathophysiology of kidney disease.

### 2.4. In-Silico Docking Analysis

For docking analysis of metabolites with the target proteins, the three-dimensional (3D) structure of various proteins was obtained from the online database RCSB Protein Data Bank (https://www.rcsb.org/pdb). The targeted proteins were named as CASP-3 (ID-3gjq) and SOD (ID-5yto), respectively.

#### 2.4.1. Ligand Preparation

The 3-D SDF file format of ligands was retrieved from the PubChem online database (https://pubchem.ncbi.nlm.nih.gov) and converted into PDB and PDBQT formats using AutoDock tool 1.5.7. and BIOVIA Discovery Studio Visualizer 2021 software, respectively. Molecular docking analysis was then performed to identify their patterns of interaction with the selected proteins. The biological interaction profile of each ligand and protein was determined successfully by adjusting torsion, degree of freedom, ionization, and stereo-chemical variation in AutoDeck [23].

#### 2.4.2. Selection of Proteins for Docking

The protein structures of CASP-3 and SOD were retrieved in PDB format with a resolution of 2.60 Å and 1.90 Å, with R values free of 0.290 and 0.215, and *R* values work 0.236 and 0.179, respectively. AutoDock 1.5.7. and BIOVIA Discovery Studio Visualizer were used to perform molecular docking analysis and visualization of the interaction between ligands and proteins. Prior to docking analysis, the commands prompt and precondition were utilized to determine the interaction energy [24].

### 2.5. ADME and Toxicity Prediction Analysis

ADME analysis of the furocoumarins found to be significantly interacting with the proteins CASP-3 and SOD, directly or indirectly, was performed to predict their pharmacokinetic behaviors. The analysis was performed using the Swiss-ADME tool as per the reference protocol with some modifications. The parameters such as lipophilicity, skin permeability index, GI absorption, blood-brain barrier index, and TEPA value of the targeted metabolites were determined. Furthermore, the toxicity profile of the targeted compounds was determined using the ProTox tool as per the reference method [25].

## 3. Results and Discussion

Quality, safety, and efficacy evaluation of medicinal plants or their active pharmaceutical ingredients are critically needed for regulatory purposes and for the scientific validation of their medicinal use. Computational techniques such as docking analysis and network pharmacology are increasingly used to establish multimechanistic therapeutic action of natural medicinal compounds against acute and chronic diseases. In this study, we evaluated the multimechanistic role of metabolites of *Apium graveolens* in the alleviation of kidney dysfunction by using computational methods.

### 3.1. Network Pharmacology Analysis

**Figure 1:**
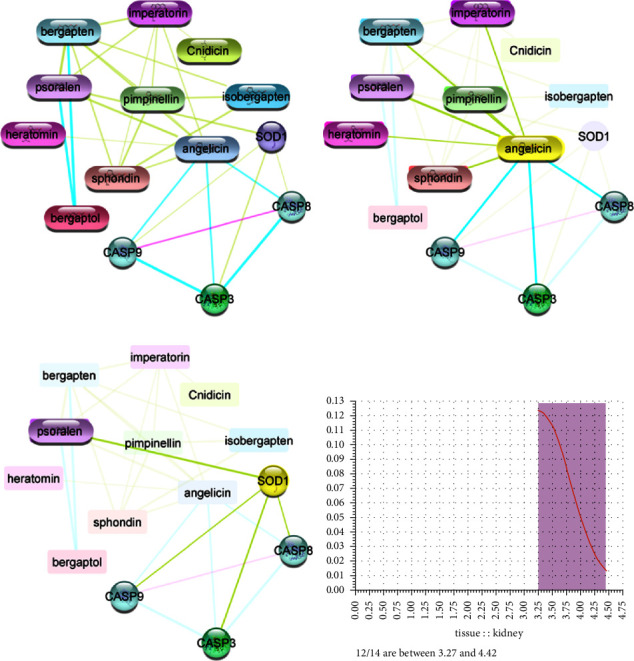
Network pharmacology analysis of furocoumarins. (a) The common active components and gene interaction. (b) Angelicin and interaction with components and genes. (c) SOD protein and its interaction with other genes and components. (d) The gene enrichment or histogram for their role in kidney dysfunction.

Several genes involved in kidney disease were screened from various public databases and used for network biology and poly-pharmacology analysis with the metabolites of *Apium graveolens.* Compound-protein interaction (CPI) network was developed to determine the interaction of metabolites with 50 genes selected for the analysis. The genes/nodes/targets that did not exhibit any interaction with the metabolites were removed during interpretation analysis. Furthermore, nontargeted interactions such as water, gases, and ions were removed during the development of the network. Only the genes with significant interactions or genes that showed at least partial interaction with the compounds were included in the final assessment. The analysis showed that a total of 5 genes interacted with the selected furocoumarin metabolites and only two metabolites, angelicin and psoralen, with CASP-3, CASP-8, CASP-9, and SOD1 proteins. Each compound node also interacted with each other in addition to the significant interaction with the genes. The compounds other than angelicin and psoralen showed no direct interaction with the genes, but they exhibited synergistic or antagonistic effects that potentiated the therapeutic effect of angelicin and psoralen. The CPI network exhibited a total of fourteen nodes and fifty edges. Each node showed significant and partial interaction with each other, showing the biological significance of these nodes. The CPI and protein-protein interaction networks are depicted in Figure 1.

Natural compounds and their derivatives are widely used as therapeutic agents for the treatment of several diseases. Currently, medicinal plants or herbal products derived from them are used as complementary and alternative therapeutic options due to the lower risk of adverse drug reactions and higher costs of modern medicines [25]. Natural products contribute significantly towards drug discovery and development [26]. Gene ontology analysis of the target genes (CASP-3, CASP-8, CASP-9, and SOD) was performed, and it was found that these genes are strongly associated with positive and negative regulation of the cellular process, signaling pathways, reduction of inflammation and stress induced by extrinsic and intrinsic stimulus, and oxidative damage. The DisGeNET analysis of the above genes showed that reperfusion injury, diabetes mellitus, hypoxia or ischemia, acute heart failure, inflammation, autoimmune disorders, myocardial reperfusion injury, and hypertension are the major pathophysiological targets that can be ameliorated by furocoumarins. Hence, from the present findings of system pharmacology, it can be suggested that furocoumarins protect kidney function by ameliorating diabetic nephropathy, hypertension, alcohol toxicity, apoptosis, and oxidative and inflammatory damage-induced renal failure. Previous studies on the pharmacological activities of *Apium graveolens* have also reported multitarget therapeutic effects of the components of this plant including antioxidant, anticancer, antidiabetic, antihypertension, anti-inflammatory, antimicrobial, and antibacterial effects against acute and chronic ailments [17, 18, 27, 28]. Kooti and Daraei reported that *Apium graveolens* plant contains many phenolic and flavonoid components such as caffeic acid, ferulic acid, p-coumaric acid, apigenin, tannin, luteolin, saponin, and kaempferol at various concentrations that significantly neutralize free radicals induced oxidative stress and act as strong antioxidant agents [18].

#### 3.1.1. Gene Ontology Analysis

**Figure 2:**
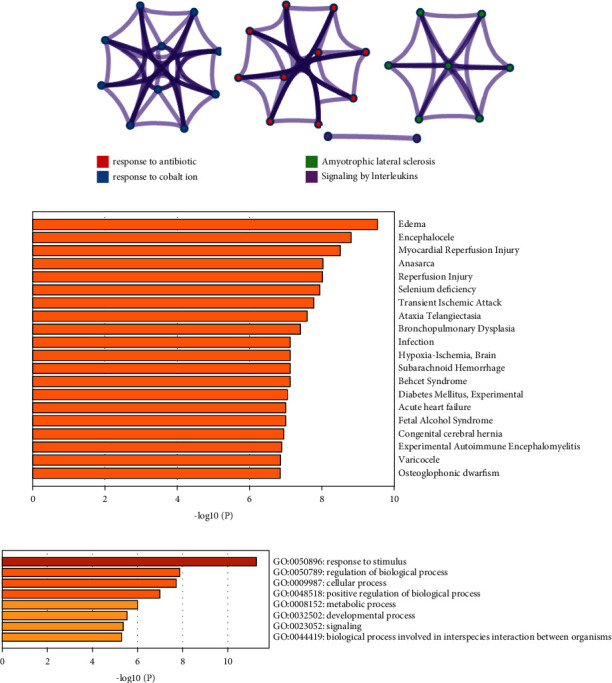
Gene ontology and disease association analysis. (a) Enrichment of gene with the pathophysiology. (b) Disease association of CASPs and SOD in different pathophysiology. (c) Different pathways are involved in the treatment of kidney disease.

Gene ontology analysis was performed to determine the multimechanistic therapeutic effects of metabolites to explore biomolecular mechanism involved in the pathophysiology of kidney disease, and the results are presented in Figure 2. It is known that CASP-3 and CASP-9 play important roles in the regulation of oxidative stress-induced inflammation, and PAMPs (nonspecific, highly conserved, pathogenic molecular structures expressed in pathogens, and their products) or DAMPs (large number of related intracellular proteins or nucleic acids released by necrotic cells at the site of necrosis) induced inflammation regulates several apoptotic and antiapoptotic pathways. In gene ontology analysis, it was found that the metabolites of *Apium graveolens* are biologically active via regulation of response to stimuli, regulation of biological positive and negative processes, cellular development process, and metabolic process, thus ameliorating reperfusion injury, diabetes mellitus, hypoxia or ischemia, acute heart failure, inflammation, autoimmune disorders, and myocardial reperfusion injury.

The previous studies suggest that renal dysfunction or kidney disease occurs due to increased vascular rigidity, hyperglycemia, inflammation-induced interstitial hemorrhage, glomerulitis, and apoptosis. In a report published by Ahmed et al., it was shown that furanocoumarins regulate several pathophysiological signaling pathways that regulate cellular apoptosis, strengthen the autophagy process against various intrinsic or extrinsic stimuli, act as antioxidants via neutralizing a variety of oxidants or free radicals, arrest cell cycle in malignant cells, and regulate metastasis, thus reducing the risk of tumor development. Additionally, furanocoumarins have also been demonstrated to have potent chemo-preventive and chemotherapeutic synergistic activities that potentiate the therapeutic effect of other antitumor drugs [29]. Ozek et al. reported a significant antioxidant effect of furocoumarins against a variety of nitrogen and oxygen free radicals that cause significant cellular morbidity [30, 31]. It has been reported that the antioxidant effect of natural components safeguards the normal function of the kidney against the oxidative stress that leads to damage renal cells or loss of kidney function [1, 8, 22].

### 3.2. In Silico Docking Analysis

**Figure 3:**
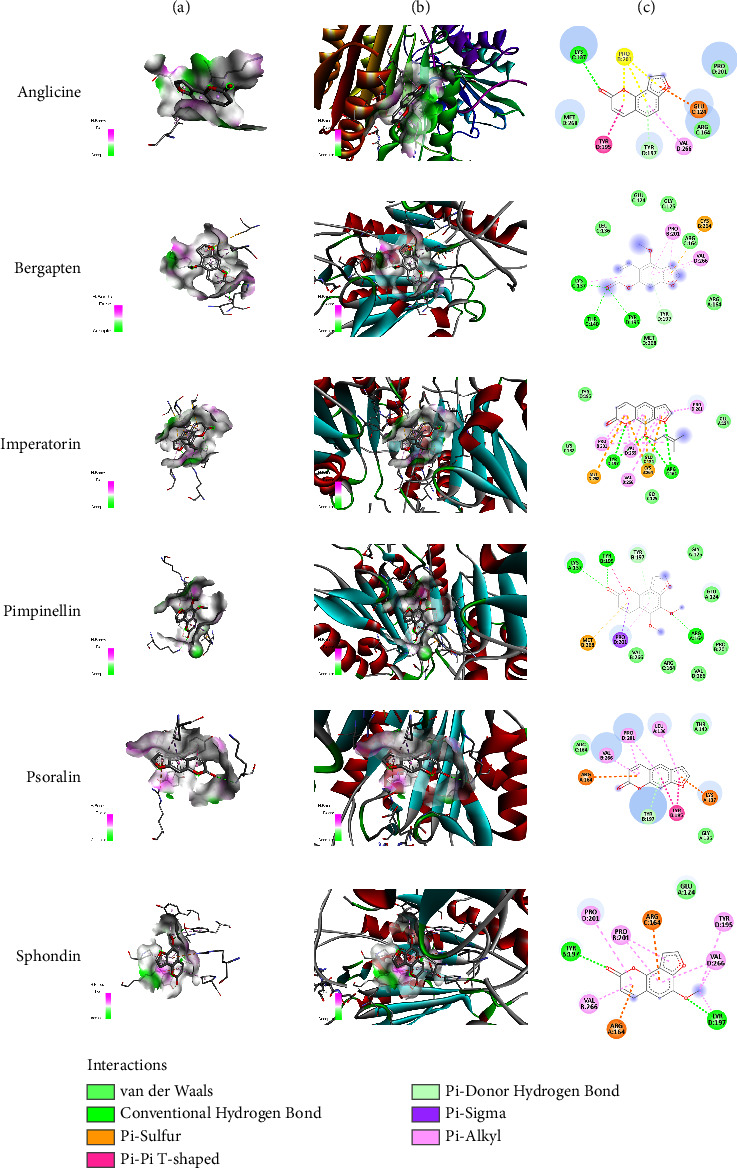
In silico docking analysis of furocoumarins with CASP-3. Column (a) represents ligand interaction with CASP-3 and column (b) represents 3D representation of ligands with protein while column (c) represents 2D representation of ligands with amino acids.

**Figure 4:**
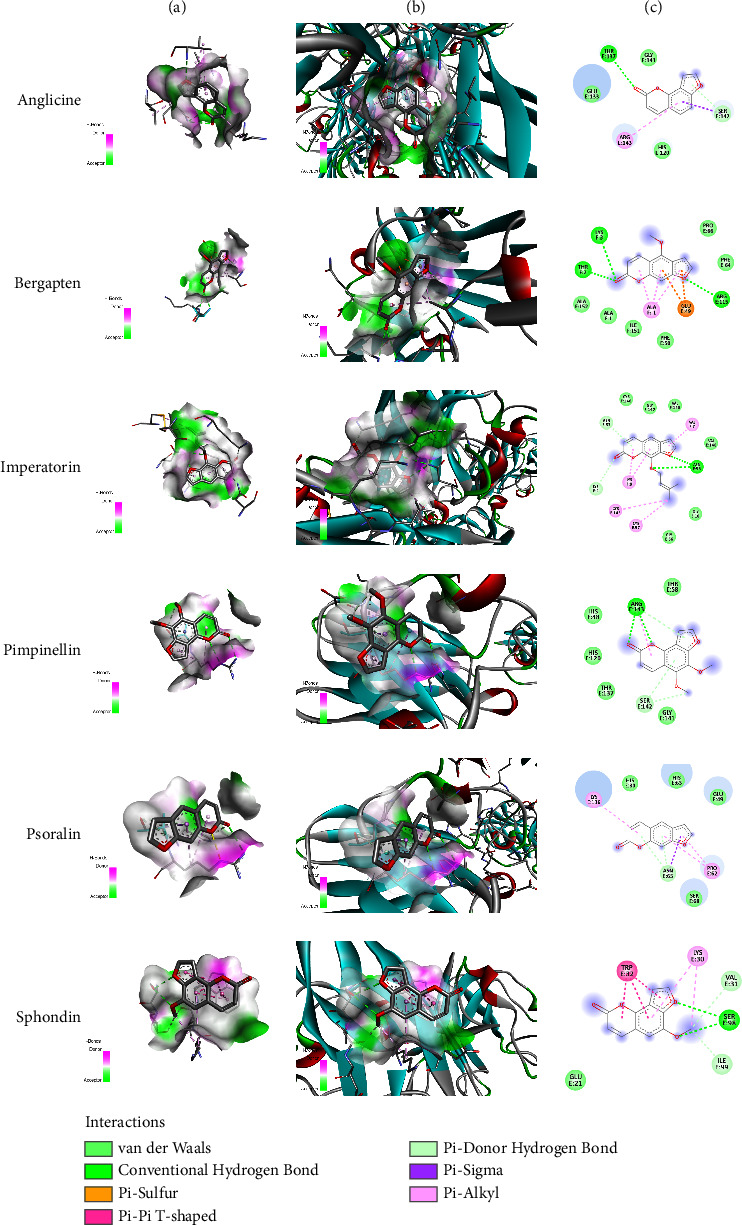
In silico docking analysis of furocoumarins with SOD. Column (a) represents ligand interaction with SOD and column (b) represents 3D representation of ligands with protein while column (c) represents 2D representation of ligands with amino acids.

In silico docking analysis was conducted on the selected genes that were found to significantly interact with the metabolites of *Apium graveolens*. In this analysis, two proteins, CASP-3 and SOD, were used to determine the interaction with furocoumarins. The interaction was determined in the form of conventional hydrogen bonds. Out of six metabolites, three metabolites, bergapten, imperatorin, and pimpinellin, exhibited significant conventional hydrogen bonding with CASP-3. The most prominent functional groups or atoms among these three compounds that interacted with CASP-3 were ketone (C=O) and ether (C-O-C) groups that produced hydrogen bonding with different amino acids. The most prominent attachments of amino acids energy pockets were found as LYS C:137, THR C: 140, and TYR D: 195 for bergapten, TYR D: 197 and ARG C: 164 for imperatorin, and LYS A: 137, TYR B: 195, and ARG A: 164 for pimpinellin, respectively. The interaction profile of SOD with the metabolites was also determined. Bergapten was found to significantly interact with SOD via conventional hydrogen bonding with amino acids LYS F: 3, THR F: 3, and ARG E: 115. Hydrogen bonds have great significance in the stability of protein-ligand complex. Several other interactions such as van der Waals, carbon-hydrogen bond, unfavorable donor-donor, Pi-cation, Pi-donor hydrogen bond, Pi-sulfur, and alkyl and Pi-alkyl bonds may negatively affect the stability of the drug-protein interactions [32]. The outcome of the in silico docking analysis is presented in Table 2 and Figures 3 and 4.

Thus, bergapten showed significant interaction with both SOD and CASP-3. Furanocoumarins ameliorate oxidative damage via regulation of the expression of xanthine oxidase (XO) and its effect on cytochrome c reduction [33]. Quetglas-Llabrés et al. reported the multimechanistic and therapeutic action of bergapten in nonalcoholic fatty liver, obesity, cancer, and bacterial infections [34]. It is suggested that in the renal failure associated with hepatic dysfunction, alcohol-induced renal failure, and bacterial-induced oxidative and inflammatory damage, bergapten can be a promising agent for treatment [8, 25, 35].

### 3.3. In Silico Pharmacokinetic Analysis

**Figure 5:**
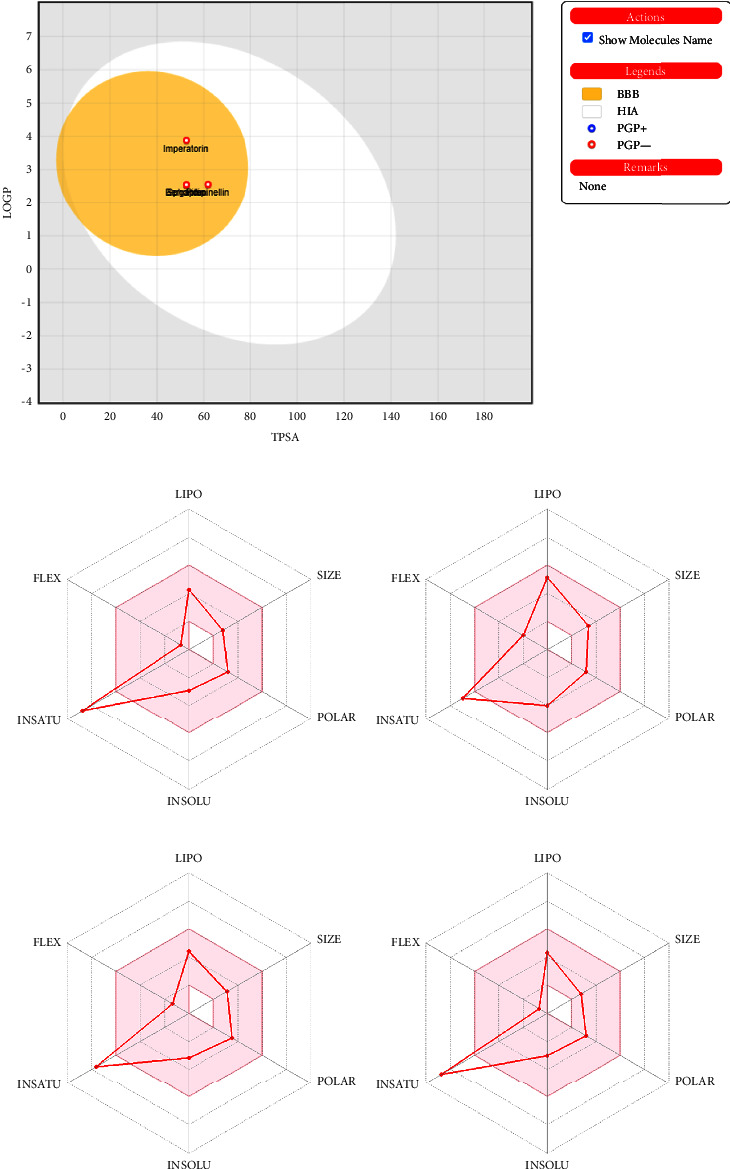
ADME analysis of targeted furocoumarins to determine their pharmacokinetic behavior. (a) represents boiled egg plots of bergapten, imperatorin, pimpinellin, and sphondin while (b–e) represents the radar plot of their physiochemical characteristic.

In silico pharmacokinetic analysis was performed to determine the pharmacokinetic behaviors of the potential metabolites interacting with SOD and CAS-3. This analysis was performed using the Swiss-ADME tool, and parameters such as lipophilicity, skin permeability, GI absorption, and blood-brain permeability index were determined to express the pharmacokinetic behavior of metabolites. The results of the study, presented in Figure 5, showed that bergapten, imperatorin, pimpinellin, and sphondin are the most lipophilic compounds that possess the consensus Log P of 2.16, 3.31, 2.22, and 2.18, respectively. A positive consensus Log *P* value represents the lipophilic character of a compound, and skin permeability index (expressed as log Kp) (cm/s) was found to be −6.25, −5.46, −6.2, and −6.16, respectively, for bergapten, imperatorin, pimpinellin, and sphondin. The greater the log Kp (cm/s) value of a component, the higher its high skin permeability. Moreover, the GI absorption and blood-brain permeability of each compound were higher than the 0.55 bioavailability score. Each compound was present in the yellow region of the egg plot which represents the high BBB permeability. The white region of the egg plot represents the low bioavailable behavior of the components.

Thus, furocoumarins have a highly lipophilic nature and high skin permeability, enabling them to easily cross the BBB and have high GI absorption. It has been reported that *Apium graveolens* contains various metabolites that can affect the therapeutic responses of other therapeutic agents. Khan et al. reported that the administration of *Apium graveolens* affects the pharmacokinetics behavior of captopril. In this study, captopril (10 mg/kg body weight (BW)) was administered orally followed by the administration of extract of the plant (40 mg/kg BW). The extract was administered one hour before the administration of captopril. The blood levels of captopril were determined using liquid chromatography-mass spectrometry (LC-MS/MS), and the parameters such as Ke, *C*_max_, *T*_max_, T1/2, and area under the curve (AUC) were determined to evaluate the pharmacokinetic behavior of captopril. The outcome of the study showed that plant extract of *Apium graveolens* increased *C*_max_ (38.67%), T1/2 (37.84%), and AUC (58.10%) and decreased Ke (27.45%) of captopril in the groups that were treated with the extract. Furthermore, it was suggested that the combination of both drugs can be a promising combination for the treatment of hypertension and its associated pathophysiological onsets including kidney disease [1, 28, 36].

The results of the present study suggest that *Apium graveolens* furocoumarins metabolites are potential anti-inflammatory and antioxidant agents for the treatment of kidney disease, as evaluated through system-based network biology and poly-pharmacology analysis.

## 4. Conclusion

Furocoumarins are one of the most promising agents in the alleviation of oxidative and inflammatory stress-induced pathophysiological morbidities including chronic kidney disease due to their ability to regulate the activity of CASP-3, CASP-8, CASP-9, and SOD. Furthermore, the biological interrelation between the furocoumarins and certain genes was also found. This system-based network biology and poly-pharmacological analysis generated enough scientific evidence for further exploration and validation of the therapeutic effect of furocoumarin-based pharmaceuticals for treating oxidative and inflammatory stress-induced kidney dysfunction and associated complications. Further experimental biomolecular approaches are necessary to enhance the credibility of the present study.

## Figures and Tables

**Table 1:** Furocoumarins present in *Apium graveolens*.

S. no	Constituents name	Chemical structure	Chemical information
1	Angelicin	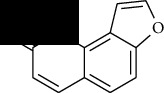	Chemical formula: C_11_H_6_O_3_
			Exact mass: 186.03
			Molecular weight: 186.17

2	Bergapten	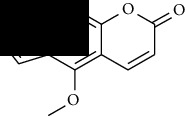	Chemical formula: C_12_H_8_O_4_
			Exact mass: 216.04
			Molecular weight: 216.19

3	Bergaptol	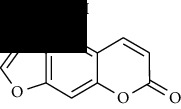	Chemical formula: C_11_H_6_O_4_
			Exact mass: 202.03
			Molecular weight: 202.17

4	Cnidicin	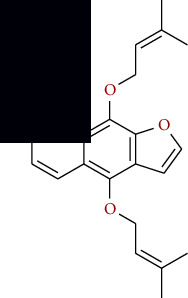	Chemical formula: C_21_H_22_O_5_
			Exact mass: 354.15
			Molecular weight: 354.40

5	Heratomin	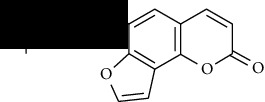	Chemical formula: C_16_H_14_O_4_
			Exact mass: 270.09
			Molecular weight: 270.28

6	Imperatorin	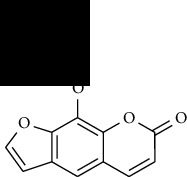	Chemical formula: C_16_H_14_O_4_
			Exact mass: 270.09
			Molecular weight: 270.28

7	Isobergapten	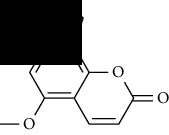	Chemical formula: C_12_H_8_O_4_
			Exact mass: 216.04
			Molecular weight: 216.19

8	Isobergaptol	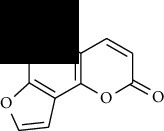	Chemical formula: C_11_H_6_O_4_
			Exact mass: 202.03
			Molecular weight: 202.17

9	Lanatin	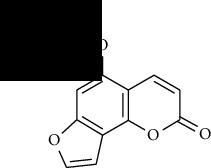	Chemical formula: C_16_H_14_O_4_
			Exact mass: 270.09
			Molecular weight: 270.28

10	Pimpinellin	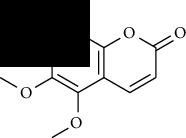	Chemical formula: C_13_H_10_O_5_
			Exact mass: 246.05
			Molecular weight: 246.22

11	Psoralen	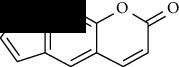	Chemical formula: C_11_H_6_O_3_
			Exact mass: 186.03
			Molecular weight: 186.17

12	Sphondin	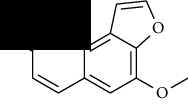	Chemical formula: C_12_H_8_O_4_
			Exact mass: 216.04
			Molecular weight: 216.19

13	Xanthotoxin	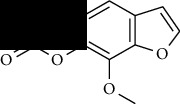	Chemical formula: C_12_H_8_O_4_
			Exact mass: 216.04
			Molecular weight: 216.19

**Table 2:** *In silico* docking analysis of furocoumarins with CASP-3 and SOD.

Compounds name	Detail of proteins					
	CASP-3			SOD		
	Grid box dimension	Binding energy (kcal/mol)	Conventional hydrogen bonding	Grid box dimension	Binding energy (kcal/mol)	Conventional hydrogen bonding
Angelicin	Center_*x* = 33.637	−6.5	LYS C: 137	Center_*x* = 62.954	−6.8	THR E: 137
Bergapten	Center_*y* = 43.345	−6.3	LYS C: 137, THR C: 140 and TYR D: 195	Center_*y* = 69.971	−5.5	LYS F: 3, THR F: 3 and ARG E: 115
Imperatorin	Center_*z* = 33.827	−7.2	TYR D: 197 and ARG C: 164	Center_*z* = 33.827	−6.2	ASN E: 53
Pimpinellin	Size_*x* = 58	−6.4	LYS A: 137, TYR B: 195 and ARG A: 164	Size_*x* = 60	−5.6	ARG E: 143
Psoralen	Size_*y* = 58	−6.2	Not found	Size_*y* = 60	−5.4	Not found
Sphondin	Size_*z* = 58	−6.5	TYR B: 197 and TYR D: 195	Size_*z* = 60	−5.7	SER E: 98

## Data Availability

The data used to support the study are included within the article.
